# The CSIESA: A Novel Score for the Assessment of Intrinsic and Extrinsic Skin Aging Based on Reflectance Confocal Microscopy Imaging

**DOI:** 10.3390/diagnostics12123161

**Published:** 2022-12-14

**Authors:** Mihai Lupu, Ana Maria Malciu, Elena Codruta Cozma, Madalina Laura Banciu, Vlad Mihai Voiculescu

**Affiliations:** 1 Department of Dermatology, Panduri Medical Center, 011367 Bucharest, Romania; 2 Department of Dermatology, “Carol Davila” University of Medicine and Pharmacy, 050474 Bucharest, Romania; 3 Department of Dermatology and Allergology, Elias Emergency University Hospital, 011461 Bucharest, Romania; 4 Department of Pathophysiology, University of Medicine and Pharmacy of Craiova, 200638 Craiova, Romania

**Keywords:** reflectance confocal microscopy, skin aging, confocal score, SCINEXA

## Abstract

Skin aging is an intricate physiological process governed by intrinsic and extrinsic factors. Increasing life expectancy has turned skin aging into a growing concern for the general population. Clinical examination of the skin does not fully describe the skin aging process. This study aims to evaluate the healthy skin of five different age groups in order to develop an easy-to-use confocal score for quantifying signs of skin aging and test the correlation between this new score and the already described clinical score, SCINEXA (score of intrinsic and extrinsic skin aging). Thirty-five subjects split into five age groups: <35; 36–45; 46–55; 56–65, and >65 years old were enrolled. Clinical signs were quantified using the SCINEXA score, and known confocal variables of skin aging were evaluated. Three different semi-quantitative scores were calculated: epidermal disarrangement score (EDS), epidermal hyperplasia score (EHS), and dermal score (DS). The EDS showed a stable trend up to the age of 65 and a dramatic increase in older subjects. EHS was characterized by an ascending trend from younger subjects to middle-aged ones. The DS was progressive with age, with a different proportion of distinct collagen types. The confocal CSIESA (confocal score for the assessment of intrinsic and extrinsic skin aging) score correlated well with the SCINEXA score. Reflectance confocal microscopy is a powerful, non-invasive technique for microscopically quantifying aging signs.

## 1. Introduction

Skin aging is an intricate physiological process that is governed by intrinsic (chronoageing) and extrinsic factors, including ultraviolet radiation exposure as one of the most important elements. Furthermore, acne and post-acne scars can negatively impact skin aging by inducing additional collagen and fat loss as well as fibrosis [1]. Increasing life expectancy and its demographic effects have turned skin aging into a topic of growing concern for the general population. This is best illustrated by the growing market for cosmetic products developed specifically to prevent, slow down or even reverse the process of skin aging. According to one online source [2], the skincare market in the United States is worth 155.8 billion US$, and it is predicted to enlarge in the upcoming years.

Recent years have shown an increase in research focused on identifying skin changes associated with aging, thus generating the need for a reliable and repeatable methodology to assess these alterations in a more diverse population [3,4]. In addition, knowledge of architectural and cellular alterations is essential for understanding and evaluating skin aging [5].

On one side, clinical examination of the skin does not fully describe the process of skin aging. Clinical scores are able to quantify only macroscopic events [6,7], usually only visible in stages of advanced alterations of the skin. In this regard, a number of descriptive skin aging scores have been developed, mainly involving photographic grading scales. These scores may be used as global indicators of skin aging [8] or photoaging [9,10].

On the other side, although skin biopsies are invasive and histological data are limited [4], the microscopic changes occurring during skin aging can now be directly observed and quantified through non-invasive techniques, such as in vivo reflectance confocal microscopy. Reflectance confocal microscopy (RCM) is a novel in vivo method which has also been applied to the study of the skin aging process [3,5,11,12,13,14,15,16,17,18,19], as it allows for a non-invasive skin examination at a quasi-histological resolution [20].

However, there are few studies that compare the microscopical features of aging skin to those of youthful skin through RCM. Therefore, our investigation aimed to study the healthy skin of five different age groups in order to develop an easy-to-use confocal score for quantifying signs of skin aging and test the correlation between this new score and the already described clinical score, SCINEXA (score of intrinsic and extrinsic skin aging).

## 2. Materials and Methods

To achieve the objectives of this investigation, we designed a prospective observational study. 

The data were collected at the “Centrul Medical Panduri” Healthcare Center in Bucharest, Romania, for six weeks, between 21 March–22 April 2022. Collected data were analyzed from 1 May 2022 to 31 June 2022. The local Institutional Review Board approved the study (protocol no. 2329/18 March 2022), and the investigation was conducted following the principles of the Declaration of Helsinki.

A formal calculation of the sample size was not performed. However, we decided to enroll in at least 30 subjects. Subjects were recruited from the in-office consultations for various dermatological concerns.

Subjects willing to participate in the study and older than six years old were included. The exclusion criteria were: history of aesthetic medicine facial procedures (e.g., blepharoplasty, botulinum toxin, micro-needling, dermabrasion, chemical peeling, laser treatments, dermal threads, PRP (Platelet-Rich Plasma) or mesotherapy treatments, or face-lift) within the last six months; application of make-up or any cosmetic product on the study area the day of the examination; active skin disease within the study area (e.g., cancer, eczema or autoimmune disease); synthetic, permanent implants in the study area; history of sun beds or intense sun-exposure in the last three months and pregnancy or breastfeeding.

Informed consent was obtained from all the subjects prior to enrollment in the study. Patient data was unlinked from personal information, anonymized, and then stored in an electronic database for subsequent statistical analysis.

### 2.1. Skin Aging Parameters

#### 2.1.1. Clinical Parameters

Clinical skin aging was quantified using the “SCINEXA” score, a validated assessment technique for skin aging, which includes items characteristic of both intrinsic (five features) and extrinsic (18 features) skin aging. The score can have a minimum of 0 (least aged) and a maximum of 69, as assessed by trained dermatologists. Each evaluated feature was graded on an ordinal scale from 0 (“none”) to 3 (“severe”). A detailed description of this score has been previously published [6].

#### 2.1.2. Reflectance Confocal Microscopy Parameters

Previously identified confocal variables related to skin aging were evaluated [13] blind from any clinical information (Table 1).

### 2.2. Description of the Confocal Score for the Assessment of Intrinsic and Extrinsic Skin Aging (CSIESA)

Working with the RCM features described in Table 1, 3 different semi-quantitative scores were calculated:

(i) Epidermal disarrangement score (range 0–12), defined as the sum of the extent of irregular honeycomb pattern (absent = 0; <10% = 1; 10–50% = 2; >50% = 3), minimal epidermal thickness, measured from the first optical section revealing the honeycomb pattern to the appearance of papillary rings (>20 μm = 0; <20 μm = 3), the furrow pattern (small rhomboidal = 0; large rhomboidal/disarranged rhomboidal/linear = 3), and the furrow width (mean of 3 random measurements) graded as 0 (narrow =< 50 μm) or 3 (wide =>50 μm);
(ii) Epidermal hyperplasia score (range 0–9), defined as the sum of the extent of mottled pigmentation (absent = 0; <10% = 1; 10–50% = 2; >50% = 3), the extent of polycyclic papillary rings (absent = 0; <10% = 1; 10–50% = 2; >50% = 3), and the full epidermal thickness (<40 μm = 0; >40 μm = 3);
(iii) Dermal score (range 0–15), defined as the sum of each collagen type area (from 0 to 4, allocating one point for every 25% of the surface covered by that specific type of collagen) multiplied by its coefficient (curled = 3, huddled = 2, coarse = 1, and thin reticulated = 0) [13,14], and the presence of longitudinal vessels defined as hyporeflective structures containing bright blood cells forming (branching) lines parallel to the surface, in still images (3 points).

The CSIESA score was calculated as the sum of these three individual scores and had a minimum of 0 (least aged) and a maximum of 36.

The following characteristics of participants were also recorded: age, sex, weight, height, smoking status, phototype, comorbidities, and chronic medication.

The study population was split into 5 age groups: group I: 35 years old and younger; group II: 36–45 years old; group III: 46–55 years old; group IV: 56–65 years old; group V: older than 65 years old.

### 2.3. Data Sources

Each subject was acclimated in a room at 22 °C for at least 20 min prior to the confocal evaluation while the clinical aging score was evaluated. The RCM imaging site was the same for every subject: 1 cm below the zygomatic process on the lateral canthal line on the left cheek. The acquisition of confocal images was performed by an expert in RCM (V.M.V.) using a standardized protocol for each volunteer: 3 × 3 mm mosaics at the superficial epidermis, mid-epidermis (stratum spinosum), dermal-epidermal junction, and papillary dermis, a 5 × 5 mm mosaic at the superficial epidermis, and a z-stack from the center of the mosaics (with a 2 μm z-change to 200 μm in depth, from the top of the stratum corneum to the superficial dermis). All the examinations were performed with patients in the supine position. 

All previously described RCM variables were assessed for each individual case by two different RCM readers (M.L. and A.M.M.) in blind, without knowledge of clinical and demographic information of subjects. Epidermal thickness was measured on z-stacks, whereas all the other parameters were evaluated on mosaics. The concepts of z-stacks and mosaics pertaining to in vivo reflectance confocal microscopy have been previously discussed [21].

### 2.4. Statistical Methods

Descriptive analysis was conducted to calculate absolute and relative frequencies for qualitative variables and mean standard deviation, and range for quantitative variables. 

Differences in epidermal thickness (quantitative data), the extension of irregular honeycomb patterns, mottled pigmentation, and polycyclic papillary contours among the age groups were analyzed using the Kruskal–Wallis nonparametric test.

The differences in the aspect, width and distance of the furrows and the type of dermal collagen in the five age groups were also evaluated using the Kruskal–Wallis nonparametric test. 

The correlation between age, SCINEXA score, confocal parameters, and confocal scores were calculated by means of Spearman’s Rho test.

All statistical analyses were performed using the SPSS software package (IBM, Armonk, NY, USA). A *p*-value lower than 0.05 was considered significant.

## 3. Results

### 3.1. Subjects Demographics

The study included 35 volunteers, whose data were all included in the final analysis (Table 2). There were 21 females and 14 males with a mean age of 49.74 ± 16.16 years (range 24–80). Only seven of the 35 participants were smokers (five females and two males). There were 16 subjects with phototype II (12 females and four males) and 19 subjects with phototype III (nine females and 10 males). Eighteen subjects had additional comorbidities, yet only 11 were administered chronic medication.

### 3.2. Skin Aging Assessment by Using the SCINEXA

**Figure 1:**
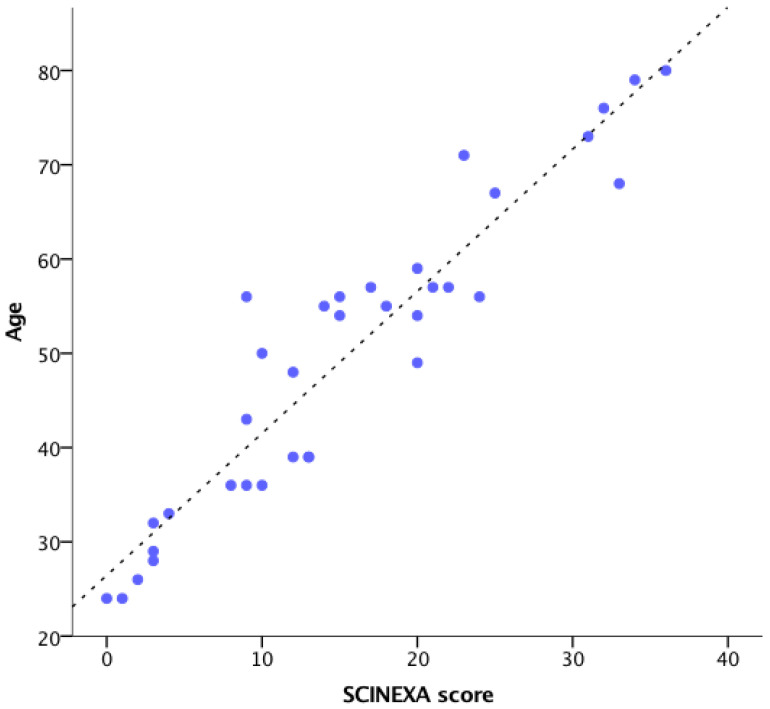
Total SCINEXA score correlated with subject age in every case.

Concerning clinical evaluation, the items characterizing the SCINEXA score, both intrinsic and extrinsic features, are shown in Table 3. This score showed an ascending trend with a good correlation with the subjects’ age (Pearson’s r = 0.932, *p* < 0.01). The total SCINEXA score was low in young participants and progressively higher in the elderly. However, a wide dispersion was observed for the SCINEXA values in the middle age groups (Figure 1). Statistically significant differences were observed only in a few age group pairs when comparing group I with group IV (H = −18.357, *p* = 0.008), group I with group V (H = −27.857, *p* = 0.000), and group II with group V (H = −19.286, *p* = 0.004).

**Figure 2:**
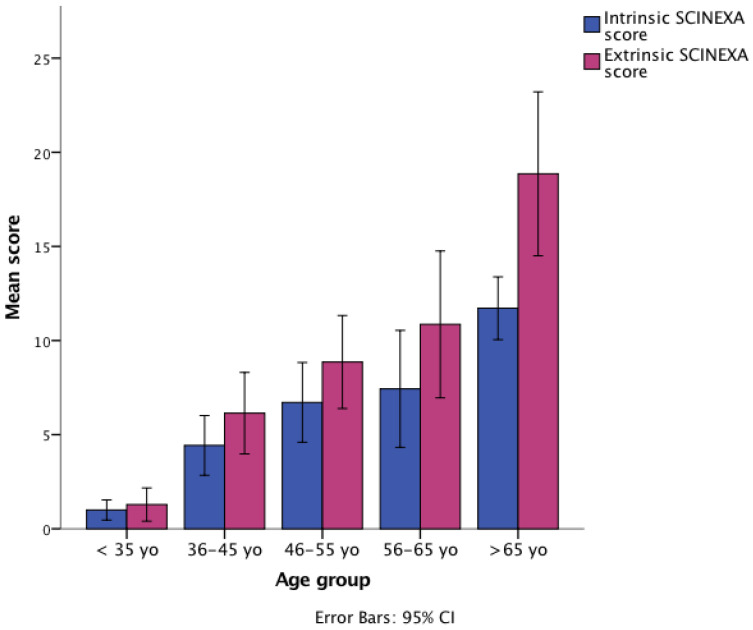
Intrinsic and extrinsic SCINEXA score differences between age groups.

When the intrinsic and extrinsic scores were considered separately, an ascending trend was observed among different age groups (*p* < 0.01; Figure 2). However, the differences were statistically significant only when comparing age group I with age groups IV and V and group II with group V. 

Concerning the individual intrinsic aging items, uneven pigmentation (*p* = 0.07), fine wrinkles (*p* = 0.02), lax appearance (*p* < 0.01), and reduced fat tissue (*p* < 0.01) differed significantly among age groups. 

For the extrinsic score items, benign skin tumors, freckles, lentigines, pigment change, change of phototype, yellowness, pseudo-scars, coarse wrinkles, elastosis, telangiectasias, and permanent erythema differed significantly (*p*-values ranging between < 0.01 and 0.035).

### 3.3. Confocal Descriptors

The frequencies of confocal descriptors in different age groups are listed in Table 4.

**Figure 3:**
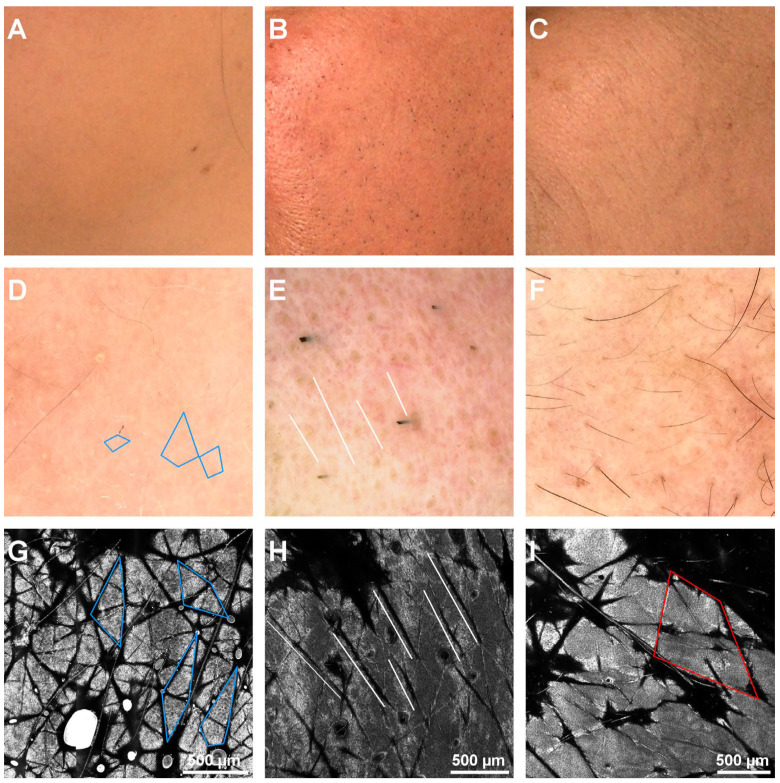
Clinical, dermoscopy, and confocal microscopy (RCM) images of different furrow patterns. (**A**). Clinical image of the left cheek of a 36-year-old female. (**B**). Clinical image of the left cheek of a 57-year-old male. (**C**). Clinical image of the left cheek of a 68-year-old female. (**D**). Dermoscopy image of the patient in panel A showing a small rhomboidal furrow pattern (blue rhombi). (**E**). Dermoscopy image of the patient in panel B showing a parallel rhomboidal furrow pattern (white lines parallel with furrows). (**F**). Dermoscopy image of the patient in panel C. (**G**). RCM mosaic showing the small rhomboidal furrow pattern (blue rhombi) in the 36-year-old female. (**H**). RCM mosaic revealing the parallel rhomboidal furrow pattern (white lines parallel with furrows) in the 57-year-old male. (**I**). RCM mosaic displaying large rhomboidal furrow pattern (red rhombus) in the 68-year-old female.

Differences in furrow patterns were observed, with the preponderance of small rhomboidal patterns in the younger subjects that were replaced with large, disarranged furrow patterns in older subjects, as seen in Table 4, with a *p* = 0.009 and H (4) = 13.53 (Kruskal-Wallis test). Figure 3 shows the confocal microscopy images of different furrow patterns. 

The furrow width between age groups was statistically significant with *p* = 0.016 and H (4) = 12.242 (Kruskal–Wallis test). The epidermis thickness was higher in young subjects and decreased in groups IV and V. Statistical significance was good with a *p* = 0.008 and H (4) = 13.707 (Kruskal–Wallis test).

The extent of an irregular honeycomb pattern, mottled pigmentation, and polycyclic papillae in different age groups is shown in Table 5.

**Figure 4:**
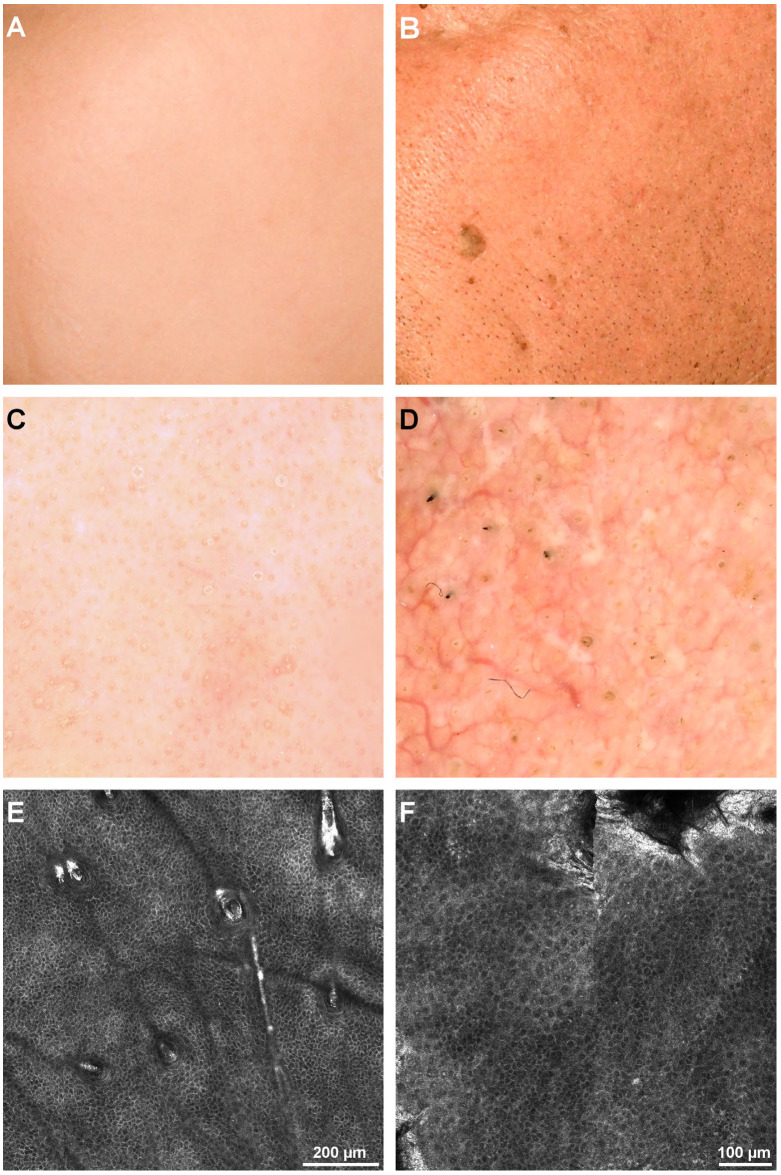
Clinical, dermoscopy, and confocal microscopy (RCM) images of honeycomb patterns. (**A**). Clinical image of left cheek skin of a 32-year-old female. (**B**). Clinical image of left cheek skin of a 76-year-old male. (**C**). Dermoscopy image of the patient in panel A showing visible pores but otherwise no noticeable alterations. (**D**). Dermoscopy image of the patient in panel B showing noticeable mottled pigmentation and several longitudinal, dilated vessels. (**E**). RCM mosaic revealing a regular honeycomb pattern in the 32-year-old female. (**F**). RCM mosaic displaying an irregular honeycomb pattern in the 76-year-old male.

Regarding the honeycomb pattern, it was observed that the percentage of irregular honeycomb patterns increased with age. Still, it wasn’t statistically significant with a *p* = 0.079 and H (4) = 8.8 (Kruskal–Wallis test). Figure 4 illustrates the RCM images of different honeycomb patterns.

**Figure 5:**
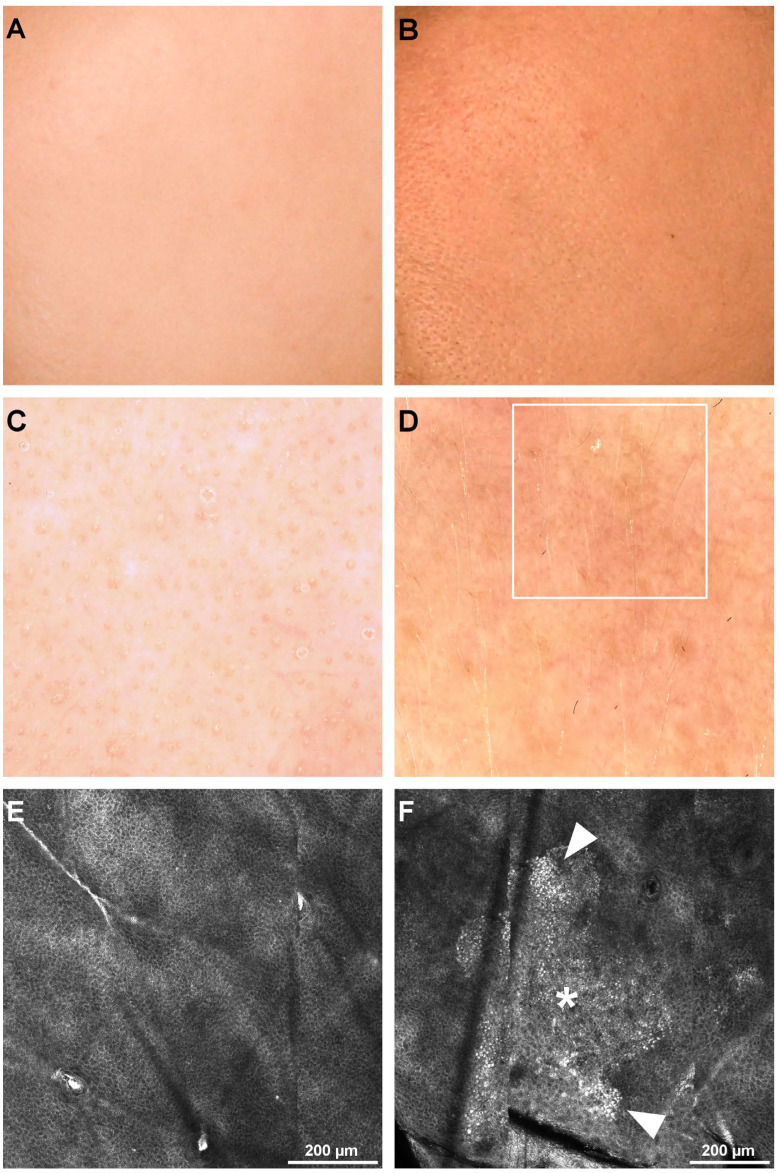
Clinical, dermoscopy, and confocal microscopy (RCM) images of honeycomb pattern with and without mottled pigmentation. (**A**). Clinical image of left cheek skin of a 32-year-old female. (**B**). Clinical image of left cheek skin of a 57-year-old female. (**C**). Dermoscopy image of the patient in panel A showing no signs of pigmentation. (**D**). Dermoscopy image of the patient in panel B revealing mottled pigmentation (white square). (**E**). RCM mosaic showing a regular honeycomb pattern without pigmentation in the 32-year-old female. (**F**). RCM mosaic displaying mottled pigmentation (white asterisk) with clusters of bright keratinocytes (white arrowheads) in the 57-year-old female.

The presence of bright keratinocytes, isolated or in clusters within the honeycomb pattern, known as mottled pigmentation, was rarely seen in groups I and II, with an increase in groups III, IV, and V. The extent of mottled pigmentation was higher (over 50%) in two subjects, one in group III and one in group V. The difference in distribution was statistically significant with *p* = 0.004 and H (4) = 15.31 (Kruskal-Wallis test). Figure 5 shows confocal microscopy images of the epidermis with and without mottled pigmentation.

**Figure 6:**
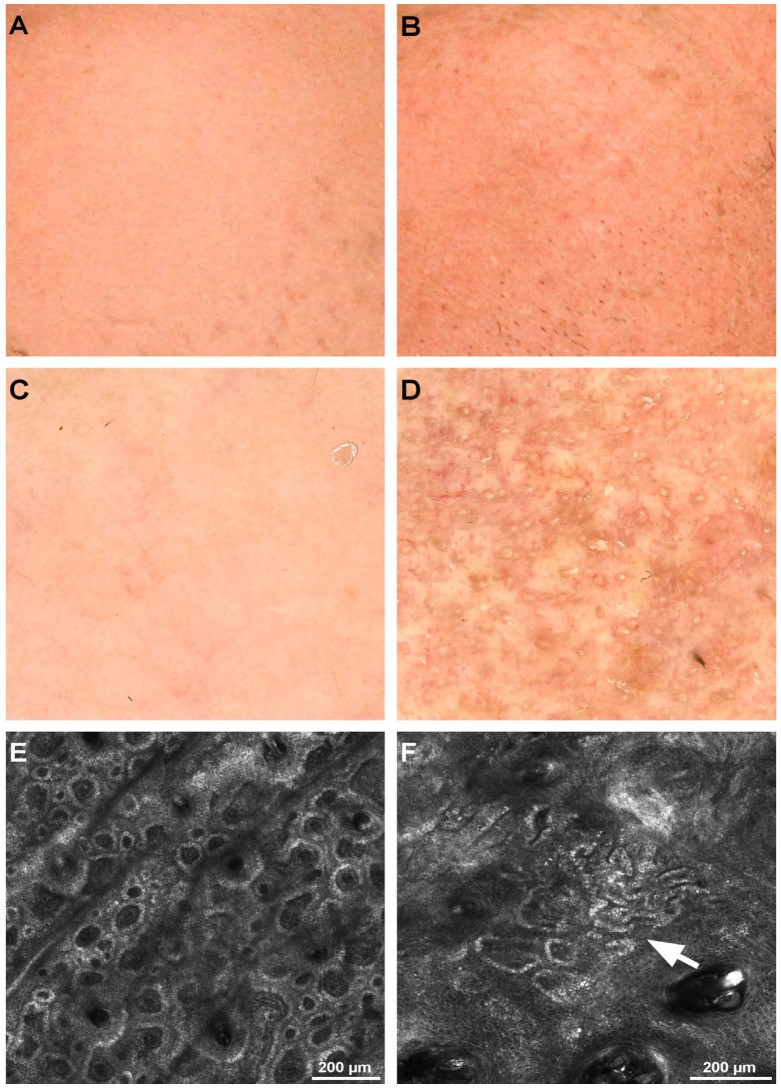
Clinical, dermoscopy, and confocal microscopy (RCM) images of regular and polycyclic papillae. (**A**). Clinical image of left cheek skin of a 36-year-old male. (**B**). Clinical image of left cheek skin of an 80-year-old male. (**C**). Dermoscopy image of the patient in panel A. (**D**). Dermoscopy image of the patient in panel B. (**E**). RCM mosaic showing regular papillae in the 36-year-old male. (**F**). RCM mosaic showing polycyclic papillae (white arrow) in the 80-year-old male.

Polycyclic papillary rings were nearly absent in the first group of participants, with all subjects in group V having elongated and partially anastomosing structures forming ring-like structures. With *p* < 0.001 and H (4) = 20.46 (Kruskal–Wallis test), the difference in distribution was statistically significant between age groups. Figure 6 depicts how these normal and polycyclic papillae appear under confocal microscopy.

**Figure 7:**
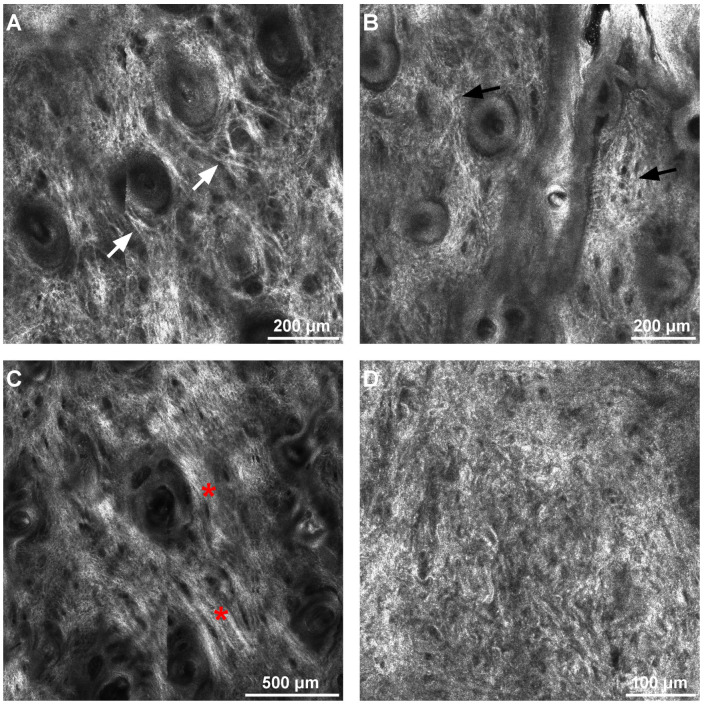
Confocal microscopy images of different types of collagen fibers. (**A**). Thin reticulated collagen fibers (white arrows) in a 28-year-old female. (**B**). Coarse collagen fibers (black arrows) in a 43-year-old male. (**C**). Huddled collagen (red asterisks) in a 57-year-old male. (**D**). Curled collagen fibers in a 68-year-old female.

**Figure 8:**
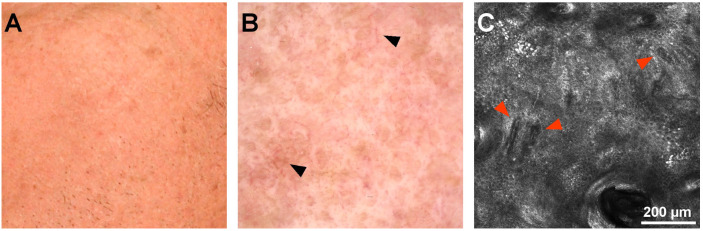
Clinical, dermoscopy, and confocal microscopy (RCM) images of left cheek skin in an 80-year-old male. (**A**). Clinical image of left cheek skin showing uneven texture and some pigmentation. (**B**). Dermoscopy image of the patient revealing pigmentation and longitudinal vessels (black arrowheads). (**C**). RCM mosaic showing longitudinal vessels (red arrowheads) in this 80-year-old male.

The dermal score is determined by the presence of different types of collagen (thin, coarse, huddled, or curled collagen fibers) and the presence of longitudinal vessels. One person can present more than one type of collagen fiber at the same time. As shown in Table 4, thin collagen fibers arranged in a reticular pattern characterized young subjects (age groups I and II) and progressively decreased in favor of coarse and huddled collagen in the remaining age groups. Huddled collagen, observable in a few cases up to group III, was notably present in groups IV and V. Curled fibers were exceedingly present in age group V, although detectable in a few instances in age groups III and IV. Figure 7 and Figure 8 show confocal microscopy images of the dermal score, which include various collagen fiber types and longitudinal vessels.

### 3.4. The CSIESA Score

The CSIESA score was calculated as the sum of three individual scores: epidermal disarrangement score, epidermal hyperplasia score, and dermal score. All three scores present significant differences for different age groups (Table 6) with a *p* < 0.05. Thus the epidermal disarrangement score has a *p* = 0.001 and an ANOVA F (4.30) = 5.88; the epidermal hyperplasia score has a *p* < 0.001 and an ANOVA F (4.30) = 8.51, and the dermal score has a *p* < 0.001 and an ANOVA F (4.30) = 21.81.

**Figure 9:**
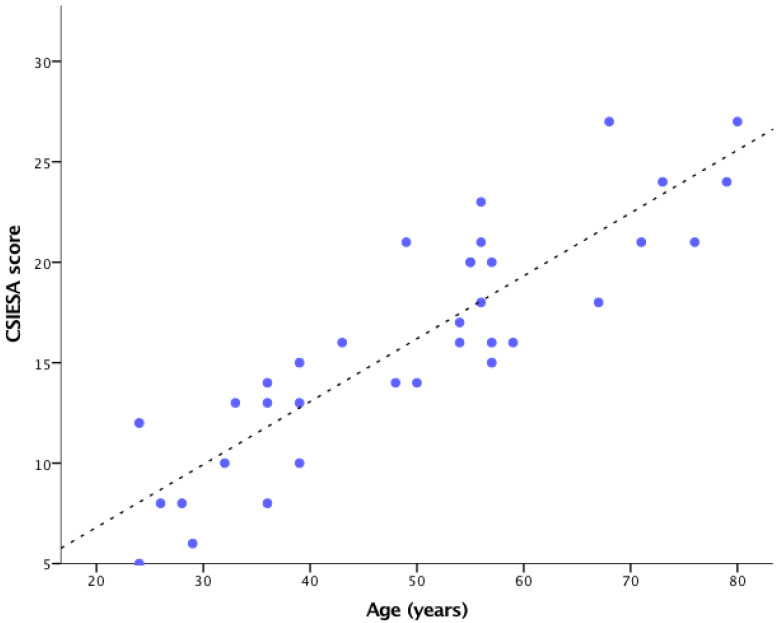
The CSIESA confocal score correlated with the age of every subject.

The CSIESA confocal score correlated very well with the subject’s age (Pearson’s r = 0.882, *p* < 0.01), as seen in Figure 9. There was a statistically significant difference in the CSIESA score between groups, as demonstrated by one-way ANOVA (F (4,30) = 23.25, *p* < 0.001). Furthermore, the upward trend in the median CSIESA score was concordant with the increase in age.

A Tukey post hoc test showed that there were statistically significant differences (*p*-value range 0.001–0.033) in the epidermal disarrangement score and epidermal hyperplasia score (as sub-components of the CSIESA score) between age group I and age groups III, IV, and V but not between age group I and II.

Dermal score values were different between group I and groups III, IV, and V (*p*-value range <0.001–0.014), between group II and groups III, IV, and V (*p*-value range <0.001–0.041), and between group V and groups I, II, and III (*p*-value range <0.001–0.001).

### 3.5. Correlations between SCINEXA Clinical Score and CSIESA Confocal Score

**Figure 10:**
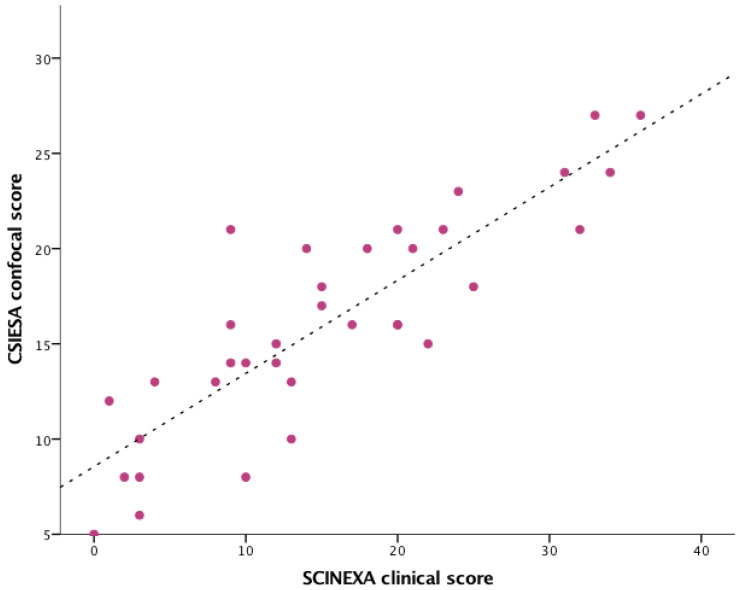
The correlation between SCINEXA clinical score and CSIESA confocal score.

The global SCINEXA clinical score that evaluates intrinsic and extrinsic aging correlated well with the confocal CSIESA score (Pearson’s r = 0.849, *p* < 0.01), as seen in Figure 10.

## 4. Discussion

Skin aging is a subject of global interest based on a complex process involving internal and external factors affecting all skin layers. The objective of our study was to develop an easy-to-use confocal score for quantifying signs of skin aging and see if this new score correlates with the already described clinical score.

In our study group, the SCINEXA clinical score increased in value simultaneously with the subject’s age, both for the overall score as well as the intrinsic and extrinsic scores.

We grouped previously described RCM parameters associated with skin aging into three semi-quantitative scores: epidermal disarrangement score (EDS), epidermal hyperplasia score (EHS), and dermal score (DS), the sum of which constitutes the CSIESA confocal score. We can distinguish between early epidermal aging (EHS) and its advanced stages (EDS) through RCM.

The epidermal thickness remains relatively constant until age 56 (due to actinic hyperplasia), then declines with age, reaching atrophy after the age of 65. The parallel furrow pattern characterizes older people, a result consistent with the specialized literature [22]. Using RCM, we have documented the progressive disorganization of the keratinocyte honeycomb pattern in the epidermis, reflecting the loss of polarization and chaotic maturation [23].

RCM can also detect pigmented foci that are not visible clinically or by dermoscopic examination. In our cohort of subjects, the mottled pigmentation covered an increasingly larger area with advancing age. This result contradicts previous studies that demonstrated a low enzymatic activity of melanocytes in the elderly [24]. However, the prevalence of lentigo maligna rises with age, making it challenging to interpret melanocyte biological changes clearly.

With time, the dermal level experiences the most severe degeneration. Young subjects presented thin reticulated fibers, which gradually changed into thick fibers, then compacted or even curved (elastosis) in elderly subjects. Without severe elastosis, collagen fibers cannot be distinguished from elastin fibers in RCM images. The aging dermis is characterized by thick, compacted or curved collagen fibers.

According to some studies, the skin has small furrow patterns that become larger and have fewer intersection lines as people age [25]. This is due to intrinsic aging, a time-dependent process based on genetic heritage and sun exposure, the most crucial external factor implicated in aging [3,26]. In our research, large rhomboidal or parallel epidermal furrows were associated with fine and coarse wrinkles, lax appearance, decreased fat tissue, and elastosis, all clinical characteristics of intrinsic and extrinsic aging. These findings are in opposition to those reported in the literature [13].

Collagen score, defined by the proportion and coefficient of coarse, huddles, and curled fibers, and irregular honeycombed pattern were found to be closely related to extrinsic SCINEXA.items such as solar lentigines, coarse wrinkles, and telangiectasias.

There was a strong connection between the presence of mottled pigmentation on the RCM examination and items of SCINEXA concerning skin pigmentation, such as uneven pigmentation, freckles, lentigines, and yellowness.

Polycyclic dermal papillae seen in the RCM images correlated well with the following SCINEXA items: lax appearance, reduced fat tissue, coarse wrinkles, and elastosis.

## 5. Conclusions

In vivo RCM is a non-invasive technique applicable to the microscopic quantification of signs of skin aging. The developed sub-scores (EDS, EHS, and DS) quantify the well-known phenomena of skin aging. The CSIESA confocal score correlated well with the SCINEXA clinical score.

The limitations of this study were the relatively small number of subjects (pilot study) and the lack of a control group in which an area without photo exposure was examined to distinguish between chronological aging and photoaging. This research will continue with the addition of new subjects and a control group.

## Figures and Tables

**Table 1:** Reflectance confocal microscopy (RCM) parameters related to skin aging [3,13].

RCM Parameter		Description
Furrow pattern [a]	Small rhomboidal	Numerous intersecting furrows forming small rhomboidal areas
	Large rhomboidal	Fewer intersecting furrows forming bigger rhomboidal areas
	Disarranged rhomboidal	Very rare intersecting furrows forming large disarranged rhomboidal areas
	Linear	Mostly parallel furrows widely spaced, which rarely intersect
Furrow width [b]	Narrow	<50 μm
	Wide	>50 μm
Honeycomb pattern [c]	Regular	Well-outlined polygonal keratinocytes, regular in size and shape
	Irregular	Pleomorphism of keratinocyte size and shape
Epidermal thickness [d]	Minimal [d1]	From the first image revealing a honeycomb pattern to the appearance of papillary rings
	Full [d2]	From the first image revealing a honeycomb pattern to the appearance of dermal collagen fibers
Mottled pigmentation [c]		Presence of bright keratinocytes, singly or in clusters, within a honeycomb pattern
Polycyclic papillary rings [c]		Elongated and partially anastomosing structures and cords forming ring-like structures
Collagen fibers [e]	Thin reticulated (×0)	Thin, reflecting fibers forming a reticulated architecture
	Coarse (×1)	Coarse, less reflecting collagen fibers forming a grossly arranged network with occasional small holes
	Huddled (×2)	Large blotches of amorphous material
	Curled (×3)	Highly refractile, thick and short wavy fibers, sometimes forming compact masses
Longitudinal vessels [a]		Hyporeflective structure containing bright blood cells forming a line parallel to the surface

[a] These features were noted as present (3) or absent (0); the presence of one pattern excluded the presence of all other furrow patterns. [b] This feature was calculated using the mean of 3 random measurements and notes as 1 for narrow and 3 for wide. [c] These features were graded on a semi-quantitative scale as follows: 0, absent; 1, <10%; 2, 10–50%; 3, >50%, of the imaged area. [d] The epidermal thickness was evaluated on a standardized z-stack with 2 μm steps, down to 100 μm in depth; [d1] 0: >20 μm, 3: <20 μm; [d2] 0: <40 μm, 3: >40 μm. [e] Each collagen type was scored from 0 to 4: 0:0%, 1:25%, 2:50%, 3:75%, and 4:75%. In the case of a smaller fraction, the remaining score point was attributed to the better-represented type. These values were calculated by multiplying each variable score (1–4) by its coefficient (×0; ×1; ×2; ×3).

**Table 2:** Demographic data.

		Female	Male
Number		21	14
Age (mean, years)		45.23	56.5
Smokers		5	2
Phototype	II	12	4
	III	9	10
Comorbidities		10	8
Chronic medication		6	5

**Table 3:** SCINEXA clinical score.

SCINEXA Score		Age Group				
		<35 Years Old (Subjects = 7)	36–45 Years Old (Subjects = 7)	46–55 Years Old (Subjects = 7)	56–65 Years Old (Subjects = 7)	>65 Years Old (Subjects = 7)
Intrinsic skin aging criteria (Number of subjects)	Uneven pigmentation	0	3	4	3	7
	Fine wrinkles	6	7	7	6	7
	Lax appearance	0	6	7	6	7
	Reduced fat tissue	0	2	7	6	7
	Benign skin tumors	1	4	5	6	7
Mean intrinsic SCINEXA score		1	4.42	6.71	7.42	11.71
Extrinsic skin aging criteria (Number of subjects)	Sunburn freckles (shoulders)	0	4	6	7	7
	Lentigines solaris (back of forearm)	1	3	6	4	7
	Pigment change	0	0	3	7	7
	Change of skin phototype	0	0	1	4	4
	Yellowness	0	1	2	3	6
	Pseudo scars	0	0	3	2	5
	Coarse wrinkles	0	4	6	6	7
	Elastosis	0	6	4	6	7
	Cutis rhomboidalis nuchae	0	0	0	1	3
	Favre racouchot	0	0	0	0	0
	Dryness (face, back of forearms)	5	4	4	6	6
	Comedones (periorbital)	0	2	1	2	3
	Telangiectasis (cheeks/nose)	3	6	6	6	6
	Permanent erythema	0	5	1	1	6
	Actinic precancerosis	0	0	0	0	1
	BCC *	0	0	0	0	1
	SCC *	0	0	0	0	0
	Melanoma	0	0	0	0	0
Mean extrinsic SCINEXA score		1.28	6.14	8.85	10.85	18.85
Mean total SCINEXA score		2.28	10.57	15.57	18.28	30.57

* BCC—basal cell carcinoma; SCC—squamous cell carcinoma.

**Table 4:** Reflectance confocal microscopy parameters related to skin aging.

		Age Group									
		<35 Years Old (Total Subjects= 7)		36–45 Years Old (Total Subjects = 7)		46–55 Years Old (Total Subjects = 7)		56–65 Years Old (Total Subjects = 7)		>65 Years Old (Total Subjects = 7)	
		Count	Mean (µm)	Count	Mean (µm)	Count	Mean (µm)	Count	Mean (µm)	Count	Mean (µm)
Furrow pattern	Small rhomboidal furrow pattern	5	-	2	-	1	-	0	-	0	-
	Large/Parallel/ Disarranged rhomboidal pattern	2	-	5	-	6	-	7	-	7	-
Furrow width			23.43		40.49		31.81		56.59		44.69
Irregular honeycomb pattern		6	-	7	-	7	-	7	-	7	-
Minimal epidermal thickness		-	45.14	-	39.43	-	42.00	-	35.43	-	32.00
Mottled pigmentation		4	-	6	-	7	-	7	-	7	-
Irregular dermal papillae		1	-	5	-	7	-	7	-	7	-
Full epidermal thickness		-	67.43	-	76.29	-	70.29	-	68.00	-	53.86
Thin collagen fibers		6	-	6	-	1	-	0	-	0	-
Coarse collagen		6	-	7	-	7	-	7	-	6	-
Huddled collagen		0	-	0	-	4	-	5	-	1	-
Curled collagen		0	-	0	-	1	-	3	-	6	-
Longitudinal vessels		0	-	2	-	3	-	1	-	3	-

**Table 5:** The extent of an irregular honeycomb pattern, mottled pigmentation, and polycyclic papillae in different age groups.

		Age Group				
		<35 Years Old (Total Subjects = 7)	36–45 Years Old (Total Subjects = 7)	46–55 Years Old (Total Subjects = 7)	56–65 Years Old (Total Subjects = 7)	>65 Years Old (Total Subjects = 7)
Irregular honeycomb pattern (extent)	0%	1	0	0	0	0
	<10%	4	4	3	6	1
	10–50%	2	3	4	1	6
Mottled pigmentation (extent)	0%	3	1	0	0	0
	<10%	3	5	3	4	0
	10–50%	1	0	3	3	6
	>50%	0	0	1	0	1
Polycyclic papillae (extent)	0%	6	2	0	0	0
	<10%	1	0	1	3	0
	10–50%	0	5	4	4	4
	>50%	0	0	2	0	3

**Table 6:** Components of CSIESA score based on age groups.

	Age Group				
	<35 Years Old (Total Subjects = 7)	36–45 Years Old (Total Subjects = 7)	46–55 Years Old (Total Subjects = 7)	56–65 Years Old (Total Subjects = 7)	>65 Years Old (Total Subjects = 7)
Epidermal disarrangement score	2	5.85	5	5.85	5.85
Epidermal hyperplasia score	3.85	5.42	6.85	5.85	5.57
Dermal score	2	2.42	6	5.28	9.57

## Data Availability

Not applicable.
